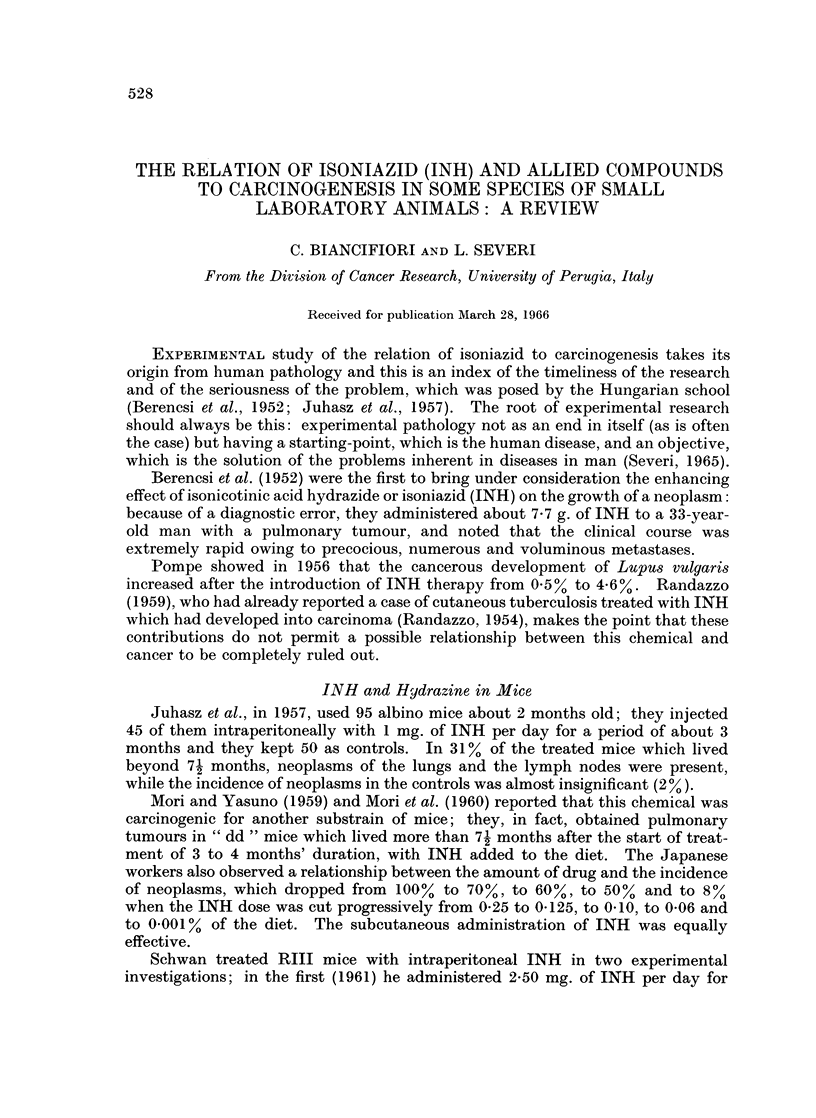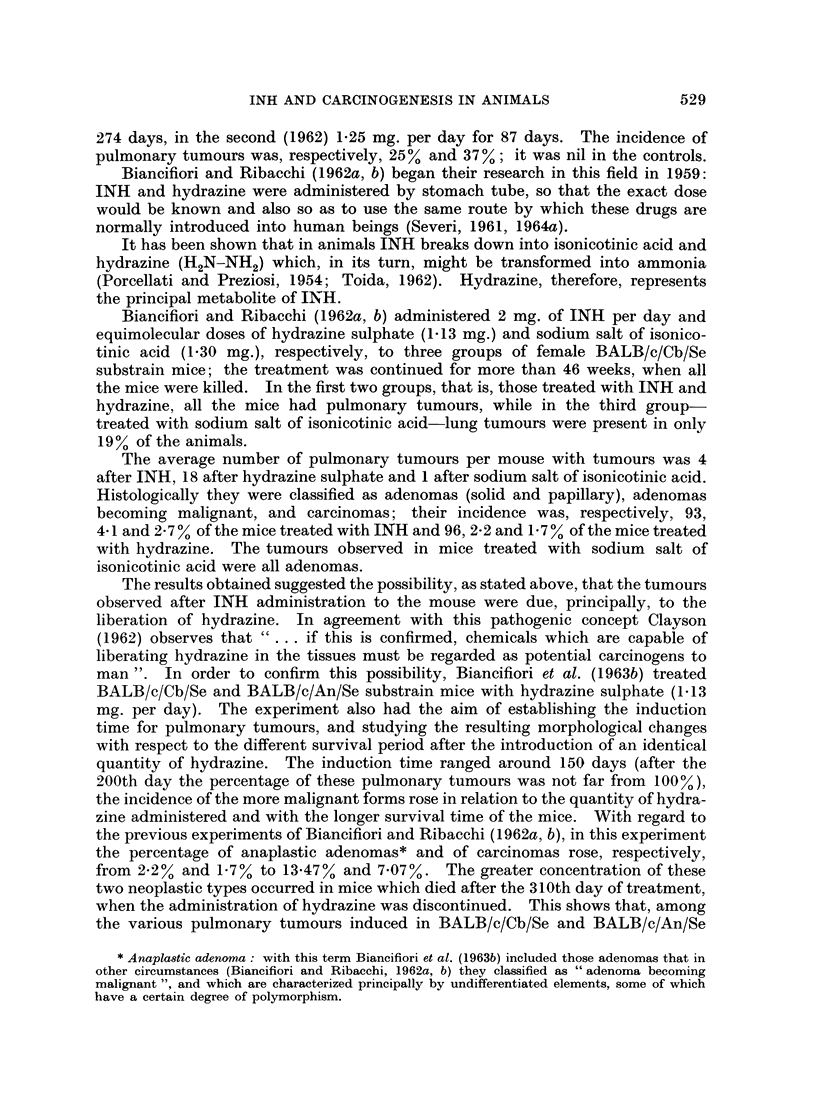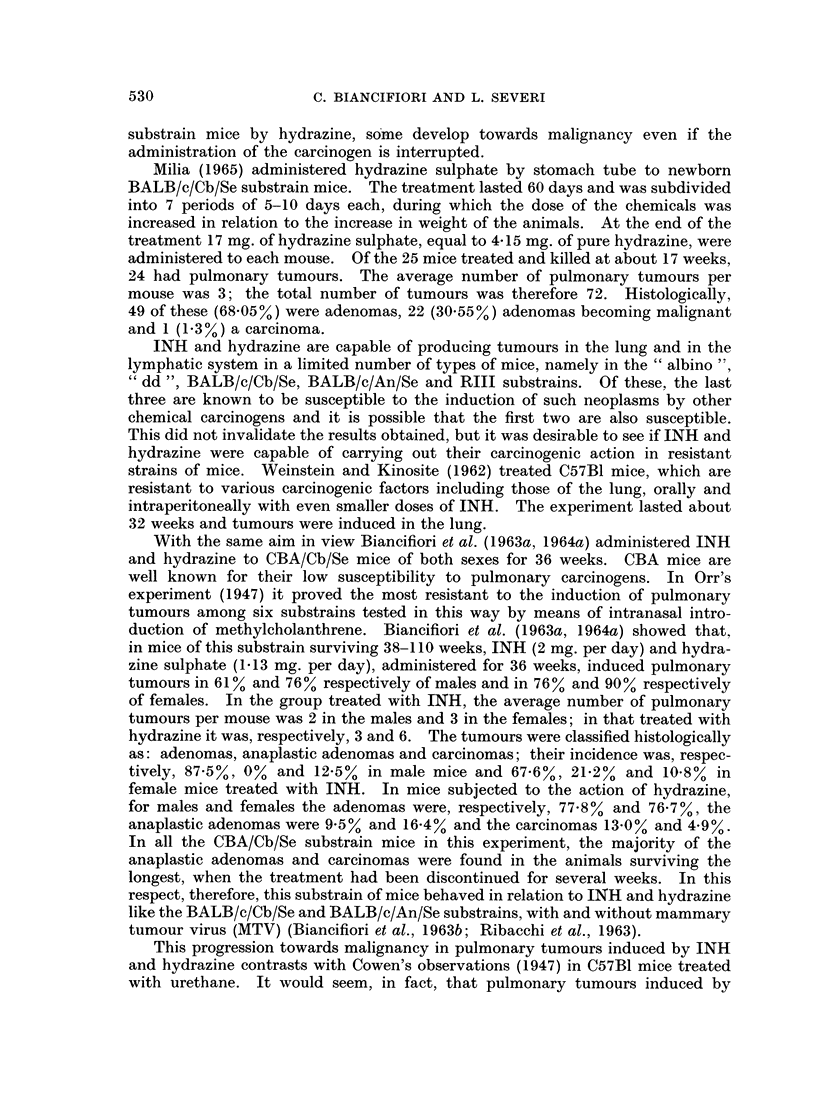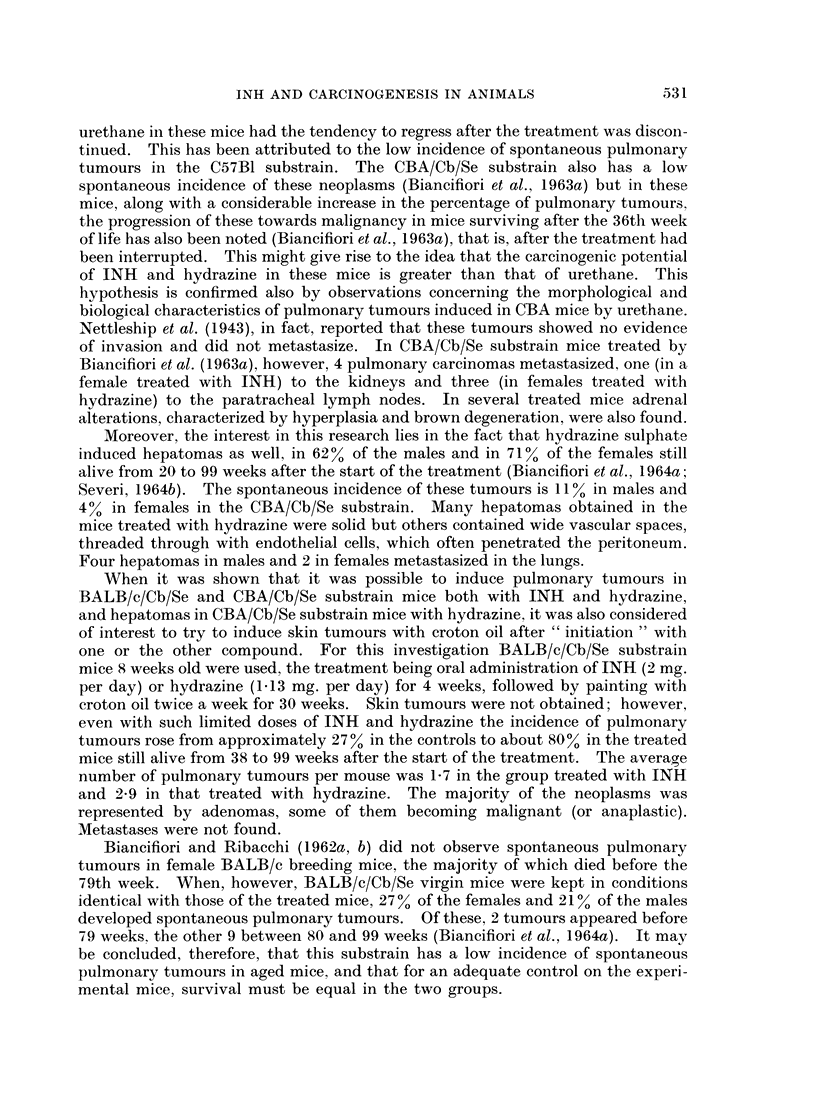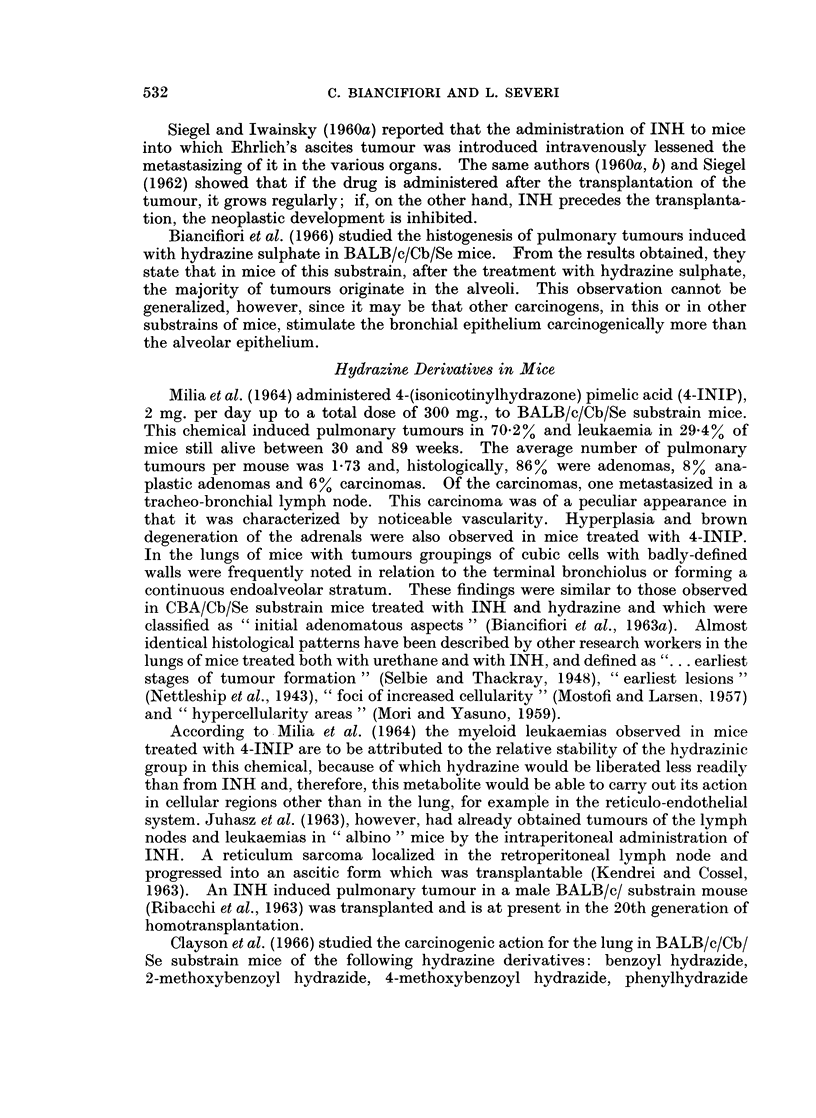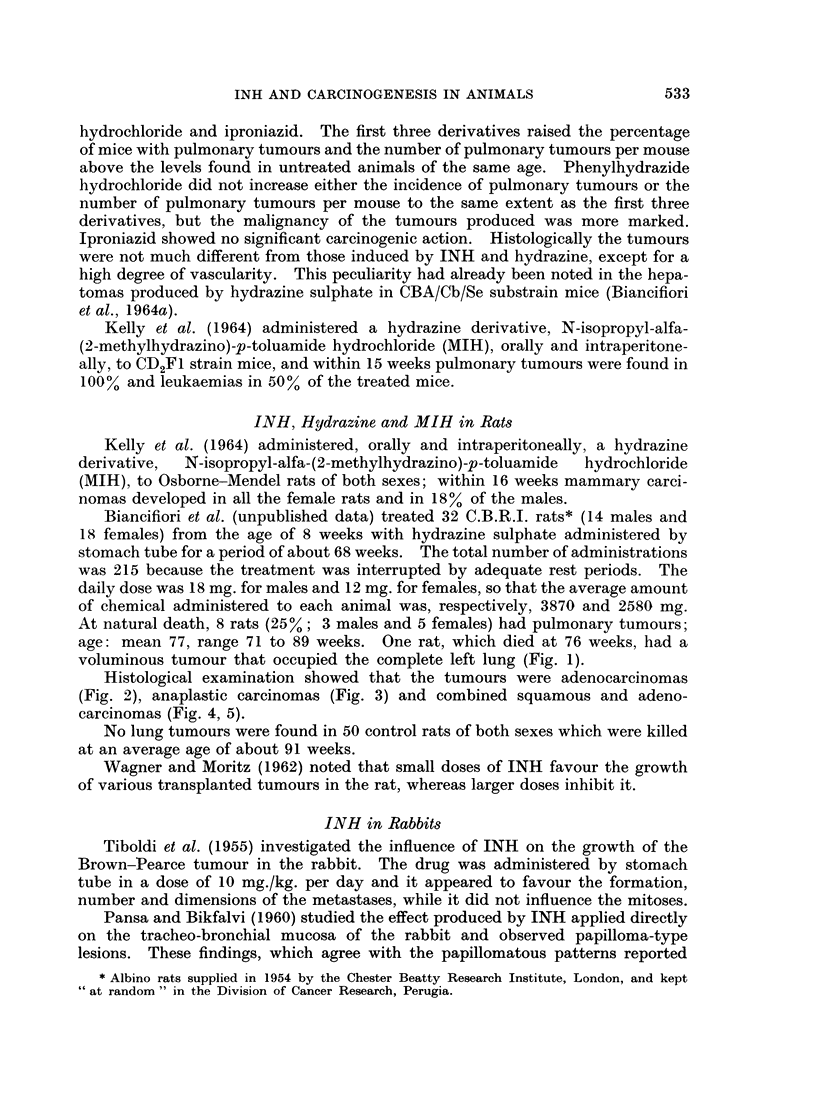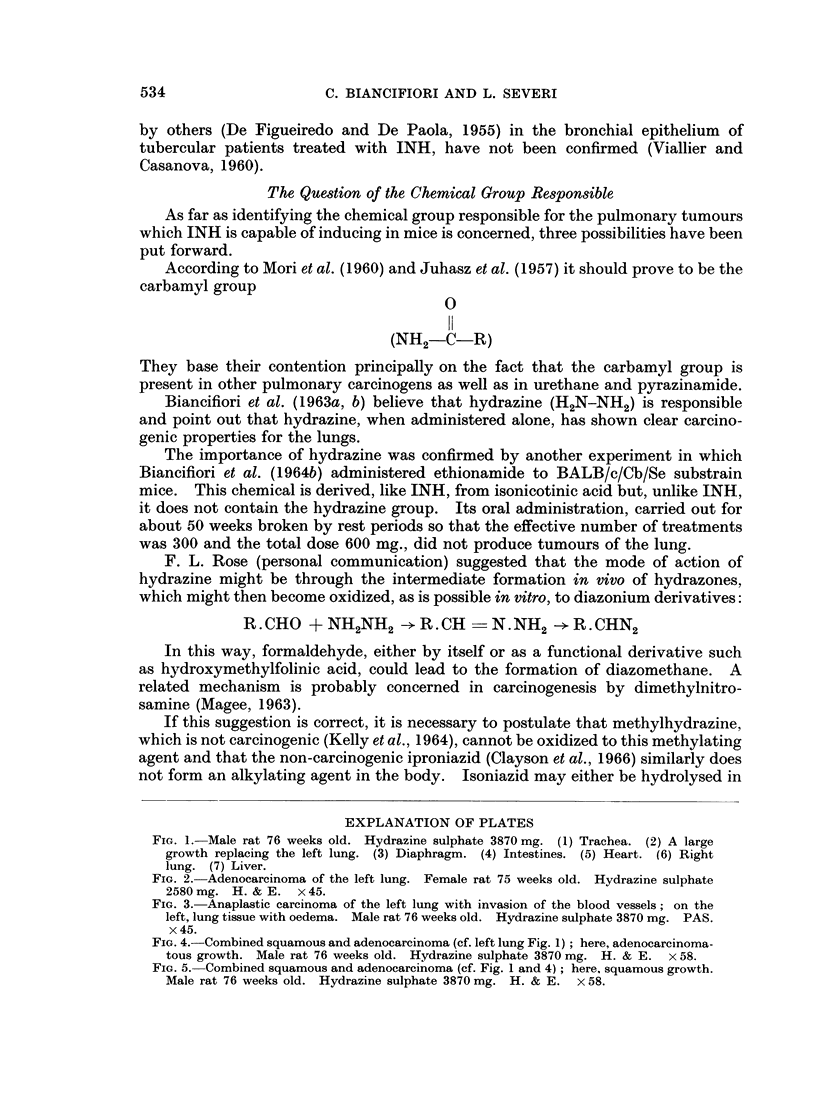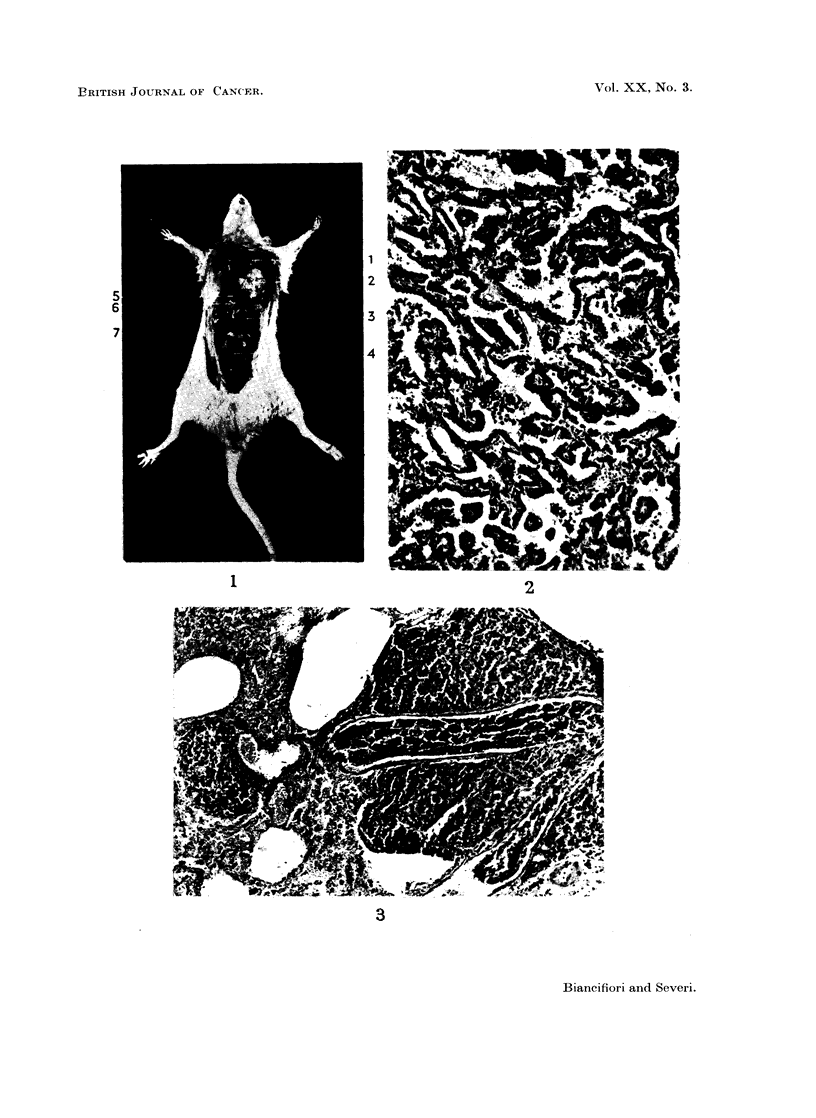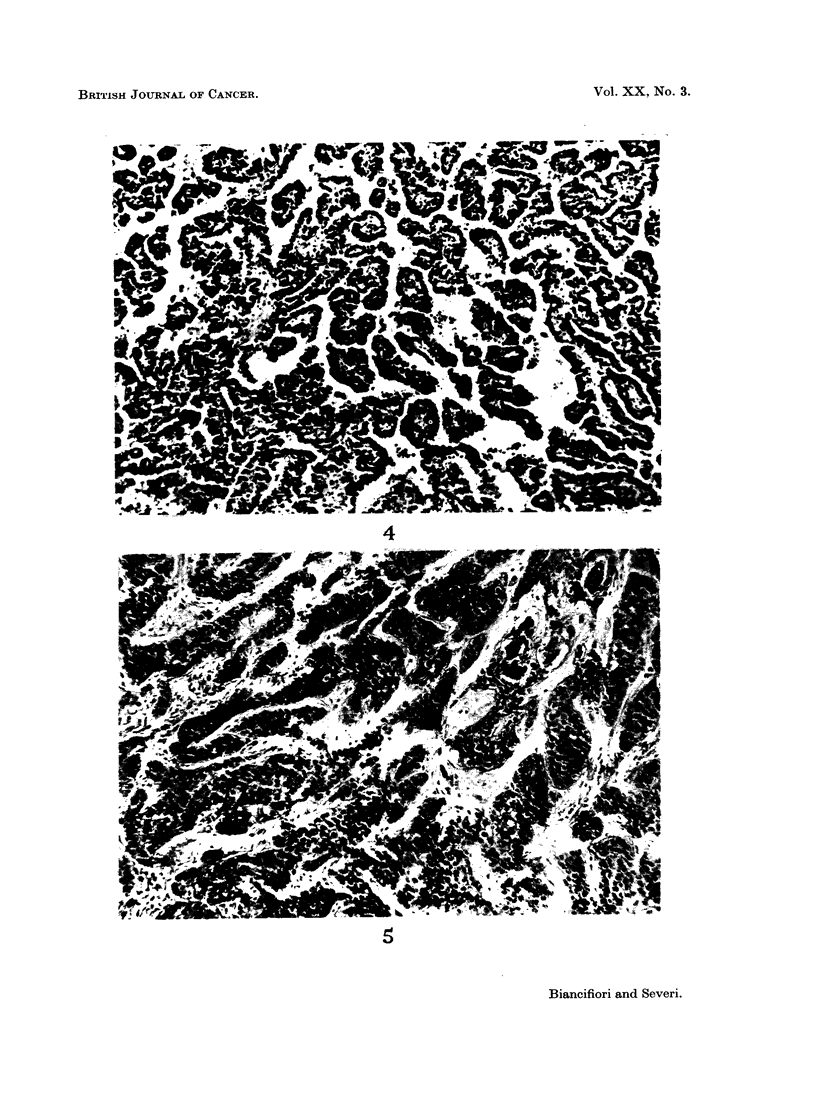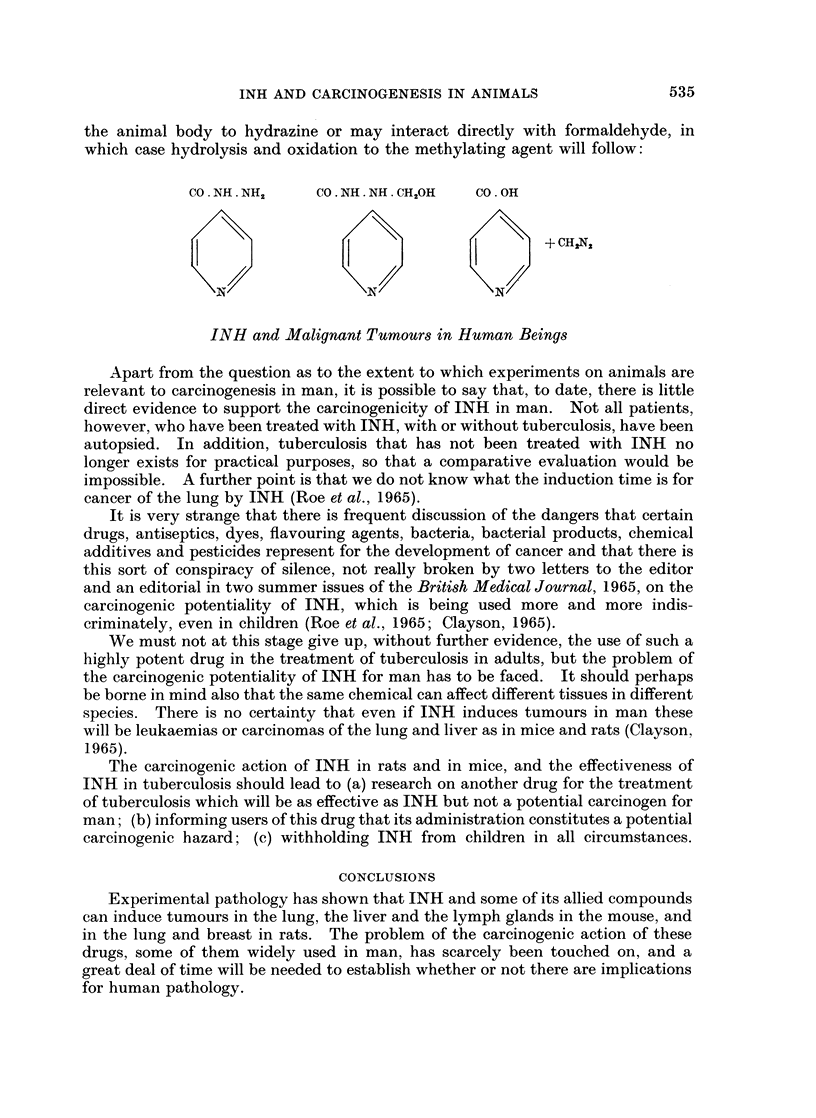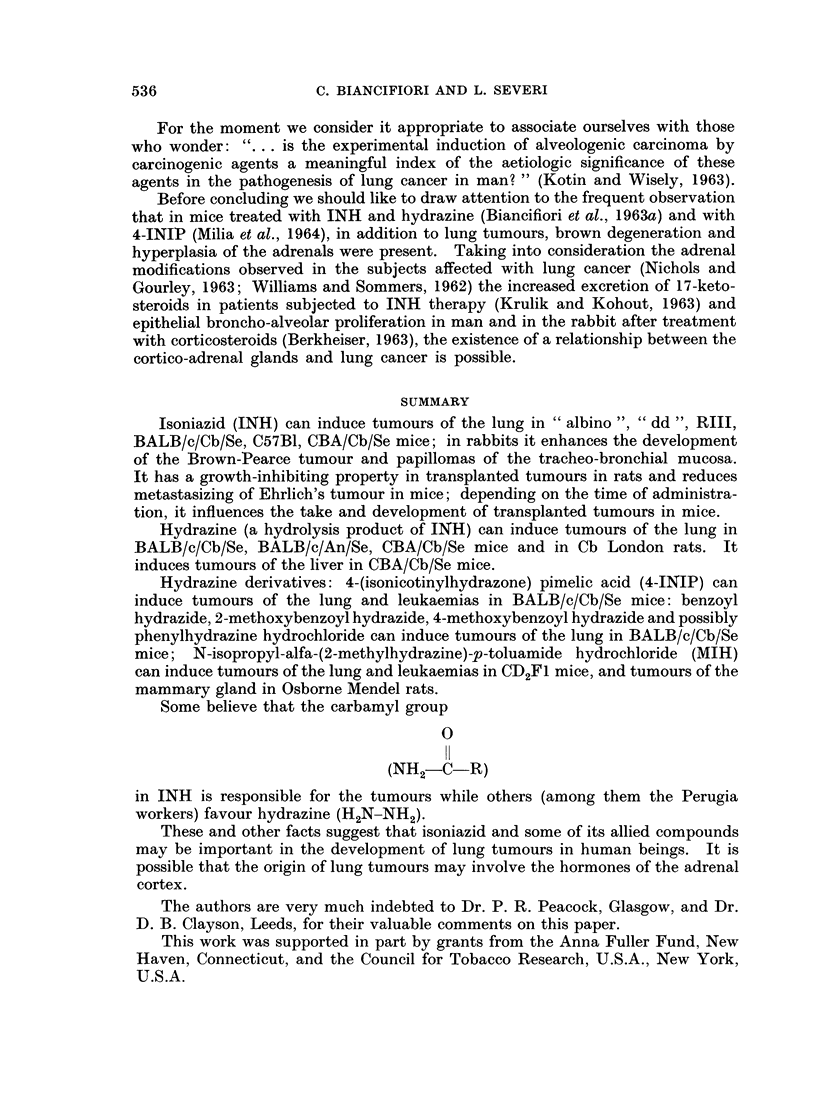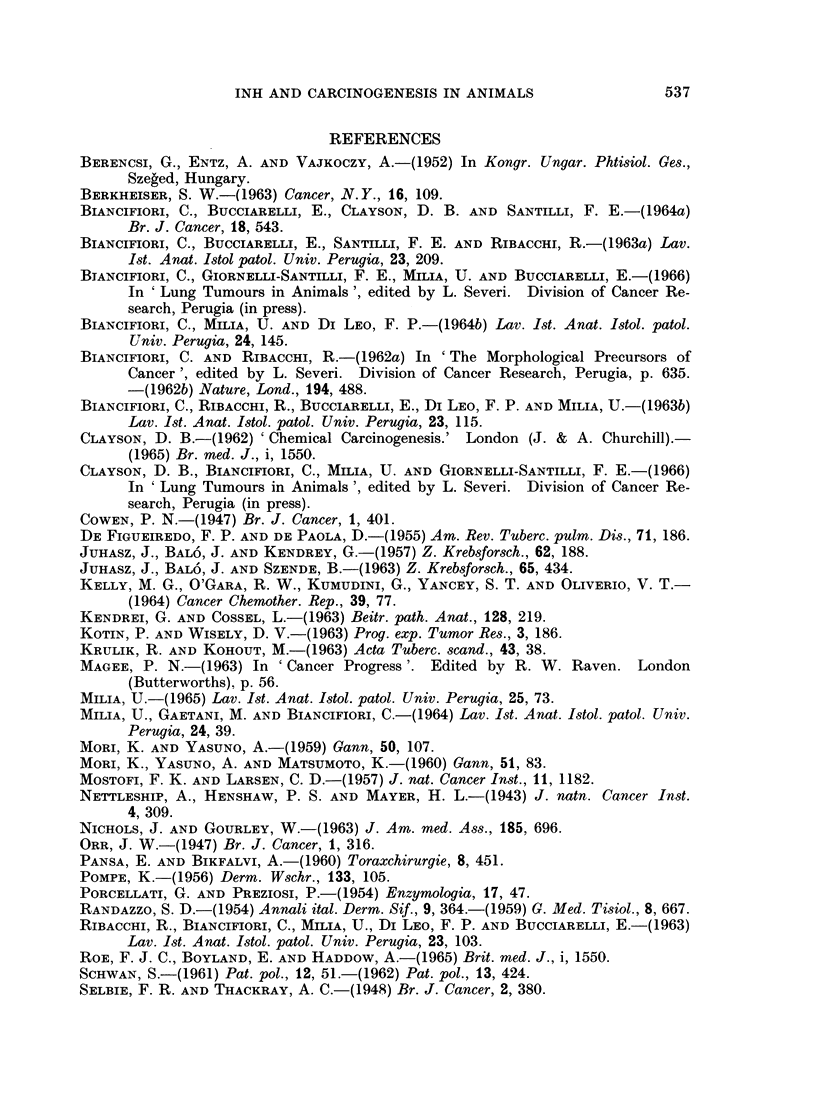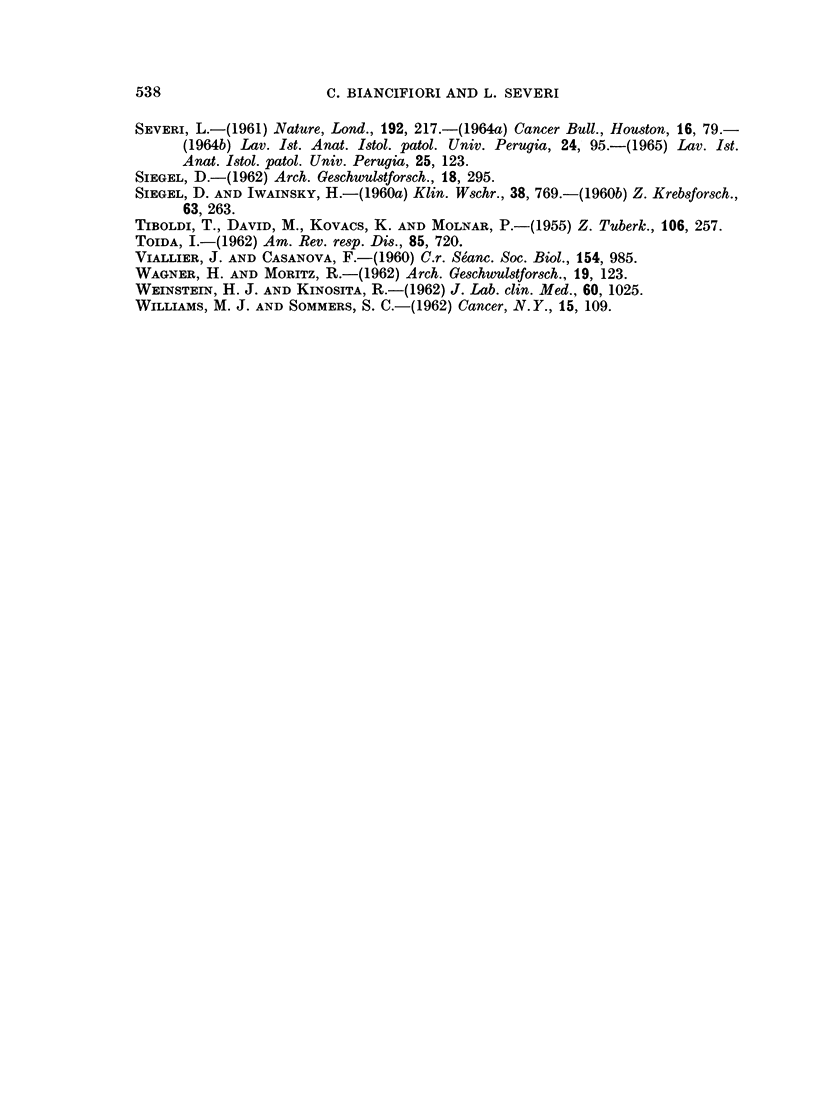# The relation of isoniazid (INH) and allied compounds to carcinogenesis in some species of small laboratory animals: a review.

**DOI:** 10.1038/bjc.1966.65

**Published:** 1966-09

**Authors:** C. Biancifiori, L. Severi

## Abstract

**Images:**


### THE RELATION OF ISONIAZID (INH) AND ALLIED COMPOUNDS TO CARCINOGENESIS IN SOME SPECIES OF SMALL LABORATORY ANIMALS: A REVIEW

C. BIANCIFIORI AND L. SEVERI

*From the Division of Cancer Research, University of Perugia, Italy*

Received for publication March 28, 1966

EXPERIMENTAL study of the relation of isoniazid to carcinogenesis takes its origin from human pathology and this is an index of the timeliness of the research and of the seriousness of the problem, which was posed by the Hungarian school (Berencsi *et al.*, 1952; Juhasz *et al.*, 1957). The root of experimental research should always be this: experimental pathology not as an end in itself (as is often the case) but having a starting-point, which is the human disease, and an objective, which is the solution of the problems inherent in diseases in man (Severi, 1965).

Berencsi *et al.* (1952) were the first to bring under consideration the enhancing effect of isonicotinic acid hydrazide or isoniazid (INH) on the growth of a neoplasm: because of a diagnostic error, they administered about 7·7 g. of INH to a 33-year-old man with a pulmonary tumour, and noted that the clinical course was extremely rapid owing to precocious, numerous and voluminous metastases.

Pompe showed in 1956 that the cancerous development of *Lupus vulgaris* increased after the introduction of INH therapy from 0·5% to 4·6%. Randazzo (1959), who had already reported a case of cutaneous tuberculosis treated with INH which had developed into carcinoma (Randazzo, 1954), makes the point that these contributions do not permit a possible relationship between this chemical and cancer to be completely ruled out.

### *INH and Hydrazine in Mice*

Juhasz *et al.*, in 1957, used 95 albino mice about 2 months old; they injected 45 of them intraperitoneally with 1 mg. of INH per day for a period of about 3 months and they kept 50 as controls. In 31% of the treated mice which lived beyond 7½ months, neoplasms of the lungs and the lymph nodes were present, while the incidence of neoplasms in the controls was almost insignificant (2%).

Mori and Yasuno (1959) and Mori *et al.* (1960) reported that this chemical was carcinogenic for another substrain of mice; they, in fact, obtained pulmonary tumours in " dd " mice which lived more than 7½ months after the start of treatment of 3 to 4 months' duration, with INH added to the diet. The Japanese workers also observed a relationship between the amount of drug and the incidence of neoplasms, which dropped from 100% to 70%, to 60%, to 50% and to 8% when the INH dose was cut progressively from 0·25 to 0·125, to 0·10, to 0·06 and to 0·001% of the diet. The subcutaneous administration of INH was equally effective.

Schwan treated RIII mice with intraperitoneal INH in two experimental investigations; in the first (1961) he administered 2·50 mg. of INH per day for

274 days, in the second (1962) 1·25 mg. per day for 87 days. The incidence of pulmonary tumours was, respectively, 25% and 37%; it was nil in the controls.

Biancifiori and Ribacchi (1962*a*, *b*) began their research in this field in 1959: INH and hydrazine were administered by stomach tube, so that the exact dose would be known and also so as to use the same route by which these drugs are normally introduced into human beings (Severi, 1961, 1964*a*).

It has been shown that in animals INH breaks down into isonicotinic acid and hydrazine ($H_2N$–$NH_2$) which, in its turn, might be transformed into ammonia (Porcellati and Preziosi, 1954; Toida, 1962). Hydrazine, therefore, represents the principal metabolite of INH.

Biancifiori and Ribacchi (1962*a*, *b*) administered 2 mg. of INH per day and equimolecular doses of hydrazine sulphate (1·13 mg.) and sodium salt of isonicotinic acid (1·30 mg.), respectively, to three groups of female BALB/c/Cb/Se substrain mice; the treatment was continued for more than 46 weeks, when all the mice were killed. In the first two groups, that is, those treated with INH and hydrazine, all the mice had pulmonary tumours, while in the third group—treated with sodium salt of isonicotinic acid—lung tumours were present in only 19% of the animals.

The average number of pulmonary tumours per mouse with tumours was 4 after INH, 18 after hydrazine sulphate and 1 after sodium salt of isonicotinic acid. Histologically they were classified as adenomas (solid and papillary), adenomas becoming malignant, and carcinomas; their incidence was, respectively, 93, 4·1 and 2·7% of the mice treated with INH and 96, 2·2 and 1·7% of the mice treated with hydrazine. The tumours observed in mice treated with sodium salt of isonicotinic acid were all adenomas.

The results obtained suggested the possibility, as stated above, that the tumours observed after INH administration to the mouse were due, principally, to the liberation of hydrazine. In agreement with this pathogenic concept Clayson (1962) observes that " . . . if this is confirmed, chemicals which are capable of liberating hydrazine in the tissues must be regarded as potential carcinogens to man ". In order to confirm this possibility, Biancifiori *et al.* (1963*b*) treated BALB/c/Cb/Se and BALB/c/An/Se substrain mice with hydrazine sulphate (1·13 mg. per day). The experiment also had the aim of establishing the induction time for pulmonary tumours, and studying the resulting morphological changes with respect to the different survival period after the introduction of an identical quantity of hydrazine. The induction time ranged around 150 days (after the 200th day the percentage of these pulmonary tumours was not far from 100%), the incidence of the more malignant forms rose in relation to the quantity of hydrazine administered and with the longer survival time of the mice. With regard to the previous experiments of Biancifiori and Ribacchi (1962*a*, *b*), in this experiment the percentage of anaplastic adenomas* and of carcinomas rose, respectively, from 2·2% and 1·7% to 13·47% and 7·07%. The greater concentration of these two neoplastic types occurred in mice which died after the 310th day of treatment, when the administration of hydrazine was discontinued. This shows that, among the various pulmonary tumours induced in BALB/c/Cb/Se and BALB/c/An/Se

* *Anaplastic adenoma* : with this term Biancifiori *et al.* (1963*b*) included those adenomas that in other circumstances (Biancifiori and Ribacchi, 1962*a*, *b*) they classified as " adenoma becoming malignant ", and which are characterized principally by undifferentiated elements, some of which have a certain degree of polymorphism.

substrain mice by hydrazine, some develop towards malignancy even if the administration of the carcinogen is interrupted.

Milia (1965) administered hydrazine sulphate by stomach tube to newborn BALB/c/Cb/Se substrain mice. The treatment lasted 60 days and was subdivided into 7 periods of 5–10 days each, during which the dose of the chemicals was increased in relation to the increase in weight of the animals. At the end of the treatment 17 mg. of hydrazine sulphate, equal to 4·15 mg. of pure hydrazine, were administered to each mouse. Of the 25 mice treated and killed at about 17 weeks, 24 had pulmonary tumours. The average number of pulmonary tumours per mouse was 3; the total number of tumours was therefore 72. Histologically, 49 of these (68·05%) were adenomas, 22 (30·55%) adenomas becoming malignant and 1 (1·3%) a carcinoma.

INH and hydrazine are capable of producing tumours in the lung and in the lymphatic system in a limited number of types of mice, namely in the " albino ", " dd ", BALB/c/Cb/Se, BALB/c/An/Se and RIII substrains. Of these, the last three are known to be susceptible to the induction of such neoplasms by other chemical carcinogens and it is possible that the first two are also susceptible. This did not invalidate the results obtained, but it was desirable to see if INH and hydrazine were capable of carrying out their carcinogenic action in resistant strains of mice. Weinstein and Kinosite (1962) treated C57Bl mice, which are resistant to various carcinogenic factors including those of the lung, orally and intraperitoneally with even smaller doses of INH. The experiment lasted about 32 weeks and tumours were induced in the lung.

With the same aim in view Biancifiori *et al.* (1963*a*, 1964*a*) administered INH and hydrazine to CBA/Cb/Se mice of both sexes for 36 weeks. CBA mice are well known for their low susceptibility to pulmonary carcinogens. In Orr's experiment (1947) it proved the most resistant to the induction of pulmonary tumours among six substrains tested in this way by means of intranasal introduction of methylcholanthrene. Biancifiori *et al.* (1963*a*, 1964*a*) showed that, in mice of this substrain surviving 38–110 weeks, INH (2 mg. per day) and hydrazine sulphate (1·13 mg. per day), administered for 36 weeks, induced pulmonary tumours in 61% and 76% respectively of males and in 76% and 90% respectively of females. In the group treated with INH, the average number of pulmonary tumours per mouse was 2 in the males and 3 in the females; in that treated with hydrazine it was, respectively, 3 and 6. The tumours were classified histologically as: adenomas, anaplastic adenomas and carcinomas; their incidence was, respectively, 87·5%, 0% and 12·5% in male mice and 67·6%, 21·2% and 10·8% in female mice treated with INH. In mice subjected to the action of hydrazine, for males and females the adenomas were, respectively, 77·8% and 76·7%, the anaplastic adenomas were 9·5% and 16·4% and the carcinomas 13·0% and 4·9%. In all the CBA/Cb/Se substrain mice in this experiment, the majority of the anaplastic adenomas and carcinomas were found in the animals surviving the longest, when the treatment had been discontinued for several weeks. In this respect, therefore, this substrain of mice behaved in relation to INH and hydrazine like the BALB/c/Cb/Se and BALB/c/An/Se substrains, with and without mammary tumour virus (MTV) (Biancifiori *et al.*, 1963*b*; Ribacchi *et al.*, 1963).

This progression towards malignancy in pulmonary tumours induced by INH and hydrazine contrasts with Cowen's observations (1947) in C57Bl mice treated with urethane. It would seem, in fact, that pulmonary tumours induced by urethane in these mice had the tendency to regress after the treatment was discontinued. This has been attributed to the low incidence of spontaneous pulmonary tumours in the C57Bl substrain. The CBA/Cb/Se substrain also has a low spontaneous incidence of these neoplasms (Biancifiori *et al.*, 1963*a*) but in these mice, along with a considerable increase in the percentage of pulmonary tumours, the progression of these towards malignancy in mice surviving after the 36th week of life has also been noted (Biancifiori *et al.*, 1963*a*), that is, after the treatment had been interrupted. This might give rise to the idea that the carcinogenic potential of INH and hydrazine in these mice is greater than that of urethane. This hypothesis is confirmed also by observations concerning the morphological and biological characteristics of pulmonary tumours induced in CBA mice by urethane. Nettleship *et al.* (1943), in fact, reported that these tumours showed no evidence of invasion and did not metastasize. In CBA/Cb/Se substrain mice treated by Biancifiori *et al.* (1963*a*), however, 4 pulmonary carcinomas metastasized, one (in a female treated with INH) to the kidneys and three (in females treated with hydrazine) to the paratracheal lymph nodes. In several treated mice adrenal alterations, characterized by hyperplasia and brown degeneration, were also found.

Moreover, the interest in this research lies in the fact that hydrazine sulphate induced hepatomas as well, in 62% of the males and in 71% of the females still alive from 20 to 99 weeks after the start of the treatment (Biancifiori *et al.*, 1964*a*; Severi, 1964*b*). The spontaneous incidence of these tumours is 11% in males and 4% in females in the CBA/Cb/Se substrain. Many hepatomas obtained in the mice treated with hydrazine were solid but others contained wide vascular spaces, threaded through with endothelial cells, which often penetrated the peritoneum. Four hepatomas in males and 2 in females metastasized in the lungs.

When it was shown that it was possible to induce pulmonary tumours in BALB/c/Cb/Se and CBA/Cb/Se substrain mice both with INH and hydrazine, and hepatomas in CBA/Cb/Se substrain mice with hydrazine, it was also considered of interest to try to induce skin tumours with croton oil after " initiation " with one or the other compound. For this investigation BALB/c/Cb/Se substrain mice 8 weeks old were used, the treatment being oral administration of INH (2 mg. per day) or hydrazine (1·13 mg. per day) for 4 weeks, followed by painting with croton oil twice a week for 30 weeks. Skin tumours were not obtained; however, even with such limited doses of INH and hydrazine the incidence of pulmonary tumours rose from approximately 27% in the controls to about 80% in the treated mice still alive from 38 to 99 weeks after the start of the treatment. The average number of pulmonary tumours per mouse was 1·7 in the group treated with INH and 2·9 in that treated with hydrazine. The majority of the neoplasms was represented by adenomas, some of them becoming malignant (or anaplastic). Metastases were not found.

Biancifiori and Ribacchi (1962*a*, *b*) did not observe spontaneous pulmonary tumours in female BALB/c breeding mice, the majority of which died before the 79th week. When, however, BALB/c/Cb/Se virgin mice were kept in conditions identical with those of the treated mice, 27% of the females and 21% of the males developed spontaneous pulmonary tumours. Of these, 2 tumours appeared before 79 weeks, the other 9 between 80 and 99 weeks (Biancifiori *et al.*, 1964*a*). It may be concluded, therefore, that this substrain has a low incidence of spontaneous pulmonary tumours in aged mice, and that for an adequate control on the experimental mice, survival must be equal in the two groups.

Siegel and Iwainsky (1960*a*) reported that the administration of INH to mice into which Ehrlich's ascites tumour was introduced intravenously lessened the metastasizing of it in the various organs. The same authors (1960*a*, *b*) and Siegel (1962) showed that if the drug is administered after the transplantation of the tumour, it grows regularly; if, on the other hand, INH precedes the transplantation, the neoplastic development is inhibited.

Biancifiori *et al.* (1966) studied the histogenesis of pulmonary tumours induced with hydrazine sulphate in BALB/c/Cb/Se mice. From the results obtained, they state that in mice of this substrain, after the treatment with hydrazine sulphate, the majority of tumours originate in the alveoli. This observation cannot be generalized, however, since it may be that other carcinogens, in this or in other substrains of mice, stimulate the bronchial epithelium carcinogenically more than the alveolar epithelium.

### *Hydrazine Derivatives in Mice*

Milia *et al.* (1964) administered 4-(isonicotinylhydrazone) pimelic acid (4-INIP), 2 mg. per day up to a total dose of 300 mg., to BALB/c/Cb/Se substrain mice. This chemical induced pulmonary tumours in 70·2% and leukaemia in 29·4% of mice still alive between 30 and 89 weeks. The average number of pulmonary tumours per mouse was 1·73 and, histologically, 86% were adenomas, 8% anaplastic adenomas and 6% carcinomas. Of the carcinomas, one metastasized in a tracheo-bronchial lymph node. This carcinoma was of a peculiar appearance in that it was characterized by noticeable vascularity. Hyperplasia and brown degeneration of the adrenals were also observed in mice treated with 4-INIP. In the lungs of mice with tumours groupings of cubic cells with badly-defined walls were frequently noted in relation to the terminal bronchiolus or forming a continuous endoalveolar stratum. These findings were similar to those observed in CBA/Cb/Se substrain mice treated with INH and hydrazine and which were classified as " initial adenomatous aspects " (Biancifiori *et al.*, 1963*a*). Almost identical histological patterns have been described by other research workers in the lungs of mice treated both with urethane and with INH, and defined as "... earliest stages of tumour formation " (Selbie and Thackray, 1948), " earliest lesions " (Nettleship *et al.*, 1943), " foci of increased cellularity " (Mostofi and Larsen, 1957) and " hypercellularity areas " (Mori and Yasuno, 1959).

According to Milia *et al.* (1964) the myeloid leukaemias observed in mice treated with 4-INIP are to be attributed to the relative stability of the hydrazinic group in this chemical, because of which hydrazine would be liberated less readily than from INH and, therefore, this metabolite would be able to carry out its action in cellular regions other than in the lung, for example in the reticulo-endothelial system. Juhasz *et al.* (1963), however, had already obtained tumours of the lymph nodes and leukaemias in " albino " mice by the intraperitoneal administration of INH. A reticulum sarcoma localized in the retroperitoneal lymph node and progressed into an ascitic form which was transplantable (Kendrei and Cossel, 1963). An INH induced pulmonary tumour in a male BALB/c/ substrain mouse (Ribacchi *et al.*, 1963) was transplanted and is at present in the 20th generation of homotransplantation.

Clayson *et al.* (1966) studied the carcinogenic action for the lung in BALB/c/Cb/Se substrain mice of the following hydrazine derivatives: benzoyl hydrazide, 2-methoxybenzoyl hydrazide, 4-methoxybenzoyl hydrazide, phenylhydrazide hydrochloride and iproniazid. The first three derivatives raised the percentage of mice with pulmonary tumours and the number of pulmonary tumours per mouse above the levels found in untreated animals of the same age. Phenylhydrazide hydrochloride did not increase either the incidence of pulmonary tumours or the number of pulmonary tumours per mouse to the same extent as the first three derivatives, but the malignancy of the tumours produced was more marked. Iproniazid showed no significant carcinogenic action. Histologically the tumours were not much different from those induced by INH and hydrazine, except for a high degree of vascularity. This peculiarity had already been noted in the hepatomas produced by hydrazine sulphate in CBA/Cb/Se substrain mice (Biancifiori *et al.*, 1964*a*).

Kelly *et al.* (1964) administered a hydrazine derivative, N-isopropyl-alfa-(2-methylhydrazino)-*p*-toluamide hydrochloride (MIH), orally and intraperitoneally, to $CD_2F1$ strain mice, and within 15 weeks pulmonary tumours were found in 100% and leukaemias in 50% of the treated mice.

### *INH, Hydrazine and MIH in Rats*

Kelly *et al.* (1964) administered, orally and intraperitoneally, a hydrazine derivative, N-isopropyl-alfa-(2-methylhydrazino)-*p*-toluamide hydrochloride (MIH), to Osborne–Mendel rats of both sexes; within 16 weeks mammary carcinomas developed in all the female rats and in 18% of the males.

Biancifiori *et al.* (unpublished data) treated 32 C.B.R.I. rats* (14 males and 18 females) from the age of 8 weeks with hydrazine sulphate administered by stomach tube for a period of about 68 weeks. The total number of administrations was 215 because the treatment was interrupted by adequate rest periods. The daily dose was 18 mg. for males and 12 mg. for females, so that the average amount of chemical administered to each animal was, respectively, 3870 and 2580 mg. At natural death, 8 rats (25%; 3 males and 5 females) had pulmonary tumours; age: mean 77, range 71 to 89 weeks. One rat, which died at 76 weeks, had a voluminous tumour that occupied the complete left lung (Fig. 1).

Histological examination showed that the tumours were adenocarcinomas (Fig. 2), anaplastic carcinomas (Fig. 3) and combined squamous and adenocarcinomas (Fig. 4, 5).

No lung tumours were found in 50 control rats of both sexes which were killed at an average age of about 91 weeks.

Wagner and Moritz (1962) noted that small doses of INH favour the growth of various transplanted tumours in the rat, whereas larger doses inhibit it.

### *INH in Rabbits*

Tiboldi *et al.* (1955) investigated the influence of INH on the growth of the Brown–Pearce tumour in the rabbit. The drug was administered by stomach tube in a dose of 10 mg./kg. per day and it appeared to favour the formation, number and dimensions of the metastases, while it did not influence the mitoses.

Pansa and Bikfalvi (1960) studied the effect produced by INH applied directly on the tracheo-bronchial mucosa of the rabbit and observed papilloma-type lesions. These findings, which agree with the papillomatous patterns reported

* Albino rats supplied in 1954 by the Chester Beatty Research Institute, London, and kept "at random" in the Division of Cancer Research, Perugia.

by others (De Figueiredo and De Paola, 1955) in the bronchial epithelium of tubercular patients treated with INH, have not been confirmed (Viallier and Casanova, 1960).

### *The Question of the Chemical Group Responsible*

As far as identifying the chemical group responsible for the pulmonary tumours which INH is capable of inducing in mice is concerned, three possibilities have been put forward.

According to Mori *et al.* (1960) and Juhasz *et al.* (1957) it should prove to be the carbamyl group

$$(NH_2\text{—}\overset{\overset{\large O}{\|}}{C}\text{—}R)$$

They base their contention principally on the fact that the carbamyl group is present in other pulmonary carcinogens as well as in urethane and pyrazinamide.

Biancifiori *et al.* (1963*a*, *b*) believe that hydrazine ($H_2N\text{–}NH_2$) is responsible and point out that hydrazine, when administered alone, has shown clear carcinogenic properties for the lungs.

The importance of hydrazine was confirmed by another experiment in which Biancifiori *et al.* (1964*b*) administered ethionamide to BALB/c/Cb/Se substrain mice. This chemical is derived, like INH, from isonicotinic acid but, unlike INH, it does not contain the hydrazine group. Its oral administration, carried out for about 50 weeks broken by rest periods so that the effective number of treatments was 300 and the total dose 600 mg., did not produce tumours of the lung.

F. L. Rose (personal communication) suggested that the mode of action of hydrazine might be through the intermediate formation *in vivo* of hydrazones, which might then become oxidized, as is possible *in vitro*, to diazonium derivatives:

$$R.CHO + NH_2NH_2 \rightarrow R.CH = N.NH_2 \rightarrow R.CHN_2$$

In this way, formaldehyde, either by itself or as a functional derivative such as hydroxymethylfolinic acid, could lead to the formation of diazomethane. A related mechanism is probably concerned in carcinogenesis by dimethylnitrosamine (Magee, 1963).

If this suggestion is correct, it is necessary to postulate that methylhydrazine, which is not carcinogenic (Kelly *et al.*, 1964), cannot be oxidized to this methylating agent and that the non-carcinogenic iproniazid (Clayson *et al.*, 1966) similarly does not form an alkylating agent in the body. Isoniazid may either be hydrolysed in

---

EXPLANATION OF PLATES

FIG. 1.—Male rat 76 weeks old. Hydrazine sulphate 3870 mg. (1) Trachea. (2) A large growth replacing the left lung. (3) Diaphragm. (4) Intestines. (5) Heart. (6) Right lung. (7) Liver.

FIG. 2.—Adenocarcinoma of the left lung. Female rat 75 weeks old. Hydrazine sulphate 2580 mg. H. & E. ×45.

FIG. 3.—Anaplastic carcinoma of the left lung with invasion of the blood vessels; on the left, lung tissue with oedema. Male rat 76 weeks old. Hydrazine sulphate 3870 mg. PAS. ×45.

FIG. 4.—Combined squamous and adenocarcinoma (cf. left lung Fig. 1); here, adenocarcinomatous growth. Male rat 76 weeks old. Hydrazine sulphate 3870 mg. H. & E. ×58.

FIG. 5.—Combined squamous and adenocarcinoma (cf. Fig. 1 and 4); here, squamous growth. Male rat 76 weeks old. Hydrazine sulphate 3870 mg. H. & E. ×58.

1

2

3

4

5

Biancifiori and Severi.

the animal body to hydrazine or may interact directly with formaldehyde, in which case hydrolysis and oxidation to the methylating agent will follow:

*INH and Malignant Tumours in Human Beings*

Apart from the question as to the extent to which experiments on animals are relevant to carcinogenesis in man, it is possible to say that, to date, there is little direct evidence to support the carcinogenicity of INH in man. Not all patients, however, who have been treated with INH, with or without tuberculosis, have been autopsied. In addition, tuberculosis that has not been treated with INH no longer exists for practical purposes, so that a comparative evaluation would be impossible. A further point is that we do not know what the induction time is for cancer of the lung by INH (Roe *et al.*, 1965).

It is very strange that there is frequent discussion of the dangers that certain drugs, antiseptics, dyes, flavouring agents, bacteria, bacterial products, chemical additives and pesticides represent for the development of cancer and that there is this sort of conspiracy of silence, not really broken by two letters to the editor and an editorial in two summer issues of the *British Medical Journal*, 1965, on the carcinogenic potentiality of INH, which is being used more and more indiscriminately, even in children (Roe *et al.*, 1965; Clayson, 1965).

We must not at this stage give up, without further evidence, the use of such a highly potent drug in the treatment of tuberculosis in adults, but the problem of the carcinogenic potentiality of INH for man has to be faced. It should perhaps be borne in mind also that the same chemical can affect different tissues in different species. There is no certainty that even if INH induces tumours in man these will be leukaemias or carcinomas of the lung and liver as in mice and rats (Clayson, 1965).

The carcinogenic action of INH in rats and in mice, and the effectiveness of INH in tuberculosis should lead to (a) research on another drug for the treatment of tuberculosis which will be as effective as INH but not a potential carcinogen for man; (b) informing users of this drug that its administration constitutes a potential carcinogenic hazard; (c) withholding INH from children in all circumstances.

### CONCLUSIONS

Experimental pathology has shown that INH and some of its allied compounds can induce tumours in the lung, the liver and the lymph glands in the mouse, and in the lung and breast in rats. The problem of the carcinogenic action of these drugs, some of them widely used in man, has scarcely been touched on, and a great deal of time will be needed to establish whether or not there are implications for human pathology.

For the moment we consider it appropriate to associate ourselves with those who wonder: "... is the experimental induction of alveologenic carcinoma by carcinogenic agents a meaningful index of the aetiologic significance of these agents in the pathogenesis of lung cancer in man?" (Kotin and Wisely, 1963).

Before concluding we should like to draw attention to the frequent observation that in mice treated with INH and hydrazine (Biancifiori *et al.*, 1963*a*) and with 4-INIP (Milia *et al.*, 1964), in addition to lung tumours, brown degeneration and hyperplasia of the adrenals were present. Taking into consideration the adrenal modifications observed in the subjects affected with lung cancer (Nichols and Gourley, 1963; Williams and Sommers, 1962) the increased excretion of 17-keto-steroids in patients subjected to INH therapy (Krulik and Kohout, 1963) and epithelial broncho-alveolar proliferation in man and in the rabbit after treatment with corticosteroids (Berkheiser, 1963), the existence of a relationship between the cortico-adrenal glands and lung cancer is possible.

### SUMMARY

Isoniazid (INH) can induce tumours of the lung in "albino", "dd", RIII, BALB/c/Cb/Se, C57Bl, CBA/Cb/Se mice; in rabbits it enhances the development of the Brown-Pearce tumour and papillomas of the tracheo-bronchial mucosa. It has a growth-inhibiting property in transplanted tumours in rats and reduces metastasizing of Ehrlich's tumour in mice; depending on the time of administration, it influences the take and development of transplanted tumours in mice.

Hydrazine (a hydrolysis product of INH) can induce tumours of the lung in BALB/c/Cb/Se, BALB/c/An/Se, CBA/Cb/Se mice and in Cb London rats. It induces tumours of the liver in CBA/Cb/Se mice.

Hydrazine derivatives: 4-(isonicotinylhydrazone) pimelic acid (4-INIP) can induce tumours of the lung and leukaemias in BALB/c/Cb/Se mice: benzoyl hydrazide, 2-methoxybenzoyl hydrazide, 4-methoxybenzoyl hydrazide and possibly phenylhydrazine hydrochloride can induce tumours of the lung in BALB/c/Cb/Se mice; N-isopropyl-alfa-(2-methylhydrazine)-*p*-toluamide hydrochloride (MIH) can induce tumours of the lung and leukaemias in $CD_2F1$ mice, and tumours of the mammary gland in Osborne Mendel rats.

Some believe that the carbamyl group

$$(NH_2—\overset{\overset{\displaystyle O}{\|}}{C}—R)$$

in INH is responsible for the tumours while others (among them the Perugia workers) favour hydrazine ($H_2N–NH_2$).

These and other facts suggest that isoniazid and some of its allied compounds may be important in the development of lung tumours in human beings. It is possible that the origin of lung tumours may involve the hormones of the adrenal cortex.

The authors are very much indebted to Dr. P. R. Peacock, Glasgow, and Dr. D. B. Clayson, Leeds, for their valuable comments on this paper.

This work was supported in part by grants from the Anna Fuller Fund, New Haven, Connecticut, and the Council for Tobacco Research, U.S.A., New York, U.S.A.

### REFERENCES

BERENCSI, G., ENTZ, A. AND VAJKOCZY, A.—(1952) In *Kongr. Ungar. Phtisiol. Ges.*, Szeğed, Hungary.
BERKHEISER, S. W.—(1963) *Cancer, N.Y.*, **16**, 109.
BIANCIFIORI, C., BUCCIARELLI, E., CLAYSON, D. B. AND SANTILLI, F. E.—(1964*a*) *Br. J. Cancer*, **18**, 543.
BIANCIFIORI, C., BUCCIARELLI, E., SANTILLI, F. E. AND RIBACCHI, R.—(1963*a*) *Lav. Ist. Anat. Istol patol. Univ. Perugia*, **23**, 209.
BIANCIFIORI, C., GIORNELLI-SANTILLI, F. E., MILIA, U. AND BUCCIARELLI, E.—(1966) In ' Lung Tumours in Animals ', edited by L. Severi. Division of Cancer Research, Perugia (in press).
BIANCIFIORI, C., MILIA, U. AND DI LEO, F. P.—(1964*b*) *Lav. Ist. Anat. Istol. patol. Univ. Perugia*, **24**, 145.
BIANCIFIORI, C. AND RIBACCHI, R.—(1962*a*) In ' The Morphological Precursors of Cancer ', edited by L. Severi. Division of Cancer Research, Perugia, p. 635. —(1962*b*) *Nature, Lond.*, **194**, 488.
BIANCIFIORI, C., RIBACCHI, R., BUCCIARELLI, E., DI LEO, F. P. AND MILIA, U.—(1963*b*) *Lav. Ist. Anat. Istol. patol. Univ. Perugia*, **23**, 115.
CLAYSON, D. B.—(1962) ' Chemical Carcinogenesis.' London (J. & A. Churchill).—(1965) *Br. med. J.*, i, 1550.
CLAYSON, D. B., BIANCIFIORI, C., MILIA, U. AND GIORNELLI-SANTILLI, F. E.—(1966) In ' Lung Tumours in Animals ', edited by L. Severi. Division of Cancer Research, Perugia (in press).
COWEN, P. N.—(1947) *Br. J. Cancer*, **1**, 401.
DE FIGUEIREDO, F. P. AND DE PAOLA, D.—(1955) *Am. Rev. Tuberc. pulm. Dis.*, **71**, 186.
JUHASZ, J., BALÓ, J. AND KENDREY, G.—(1957) *Z. Krebsforsch.*, **62**, 188.
JUHASZ, J., BALÓ, J. AND SZENDE, B.—(1963) *Z. Krebsforsch.*, **65**, 434.
KELLY, M. G., O'GARA, R. W., KUMUDINI, G., YANCEY, S. T. AND OLIVERIO, V. T.—(1964) *Cancer Chemother. Rep.*, **39**, 77.
KENDREI, G. AND COSSEL, L.—(1963) *Beitr. path. Anat.*, **128**, 219.
KOTIN, P. AND WISELY, D. V.—(1963) *Prog. exp. Tumor Res.*, **3**, 186.
KRULIK, R. AND KOHOUT, M.—(1963) *Acta Tuberc. scand.*, **43**, 38.
MAGEE, P. N.—(1963) In ' Cancer Progress '. Edited by R. W. Raven. London (Butterworths), p. 56.
MILIA, U.—(1965) *Lav. Ist. Anat. Istol. patol. Univ. Perugia*, **25**, 73.
MILIA, U., GAETANI, M. AND BIANCIFIORI, C.—(1964) *Lav. Ist. Anat. Istol. patol. Univ. Perugia*, **24**, 39.
MORI, K. AND YASUNO, A.—(1959) *Gann*, **50**, 107.
MORI, K., YASUNO, A. AND MATSUMOTO, K.—(1960) *Gann*, **51**, 83.
MOSTOFI, F. K. AND LARSEN, C. D.—(1957) *J. nat. Cancer Inst.*, **11**, 1182.
NETTLESHIP, A., HENSHAW, P. S. AND MAYER, H. L.—(1943) *J. natn. Cancer Inst.* **4**, 309.
NICHOLS, J. AND GOURLEY, W.—(1963) *J. Am. med. Ass.*, **185**, 696.
ORR, J. W.—(1947) *Br. J. Cancer*, **1**, 316.
PANSA, E. AND BIKFALVI, A.—(1960) *Toraxchirurgie*, **8**, 451.
POMPE, K.—(1956) *Derm. Wschr.*, **133**, 105.
PORCELLATI, G. AND PREZIOSI, P.—(1954) *Enzymologia*, **17**, 47.
RANDAZZO, S. D.—(1954) *Annali ital. Derm. Sif.*, **9**, 364.—(1959) *G. Med. Tisiol.*, **8**, 667.
RIBACCHI, R., BIANCIFIORI, C., MILIA, U., DI LEO, F. P. AND BUCCIARELLI, E.—(1963) *Lav. Ist. Anat. Istol. patol. Univ. Perugia*, **23**, 103.
ROE, F. J. C., BOYLAND, E. AND HADDOW, A.—(1965) *Brit. med. J.*, i, 1550.
SCHWAN, S.—(1961) *Pat. pol.*, **12**, 51.—(1962) *Pat. pol.*, **13**, 424.
SELBIE, F. R. AND THACKRAY, A. C.—(1948) *Br. J. Cancer*, **2**, 380.

SEVERI, L.—(1961) *Nature, Lond.*, **192**, 217.—(1964*a*) *Cancer Bull., Houston*, **16**, 79.—(1964*b*) *Lav. Ist. Anat. Istol. patol. Univ. Perugia*, **24**, 95.—(1965) *Lav. Ist. Anat. Istol. patol. Univ. Perugia*, **25**, 123.
SIEGEL, D.—(1962) *Arch. Geschwulstforsch.*, **18**, 295.
SIEGEL, D. AND IWAINSKY, H.—(1960*a*) *Klin. Wschr.*, **38**, 769.—(1960*b*) *Z. Krebsforsch.*, **63**, 263.
TIBOLDI, T., DAVID, M., KOVACS, K. AND MOLNAR, P.—(1955) *Z. Tuberk.*, **106**, 257.
TOIDA, I.—(1962) *Am. Rev. resp. Dis.*, **85**, 720.
VIALLIER, J. AND CASANOVA, F.—(1960) *C.r. Séanc. Soc. Biol.*, **154**, 985.
WAGNER, H. AND MORITZ, R.—(1962) *Arch. Geschwulstforsch.*, **19**, 123.
WEINSTEIN, H. J. AND KINOSITA, R.—(1962) *J. Lab. clin. Med.*, **60**, 1025.
WILLIAMS, M. J. AND SOMMERS, S. C.—(1962) *Cancer, N.Y.*, **15**, 109.